# Development and Evaluation of a Next-Generation Sequencing Panel for the Multiple Detection and Identification of Pathogens in Fermented Foods

**DOI:** 10.4014/jmb.2211.11009

**Published:** 2022-11-16

**Authors:** Dong-Geun Park, Eun-Su Ha, Byungcheol Kang, Iseul Choi, Jeong-Eun Kwak, Jinho Choi, Jeongwoong Park, Woojung Lee, Seung Hwan Kim, Soon Han Kim, Ju-Hoon Lee

**Affiliations:** 1 Department of Food and Animal Biotechnology, Department of Agricultural Biotechnology, Research Institute of Agriculture and Life Sciences, Center for Food and Bioconvergence, Seoul National University, Seoul 08826, Republic of Korea; 2 Research and Development Center, Sanigen Co., Ltd, Anyang 14059, Republic of Korea; 3 Division of Food Microbiology, National Institute of Food and Drug Safety Evaluation, Ministry of Food and Drug Safety, Cheongju 28159, Republic of Korea

**Keywords:** NGS panel, multiple detection, foodborne pathogens, qPCR validation

## Abstract

These days, bacterial detection methods have some limitations in sensitivity, specificity, and multiple detection. To overcome these, novel detection and identification method is necessary to be developed. Recently, NGS panel method has been suggested to screen, detect, and even identify specific foodborne pathogens in one reaction. In this study, new NGS panel primer sets were developed to target 13 specific virulence factor genes from five types of pathogenic *Escherichia coli*, *Listeria monocytogenes*, and *Salmonella enterica* serovar Typhimurium, respectively. Evaluation of the primer sets using singleplex PCR, crosscheck PCR and multiplex PCR revealed high specificity and selectivity without interference of primers or genomic DNAs. Subsequent NGS panel analysis with six artificially contaminated food samples using those primer sets showed that all target genes were multi-detected in one reaction at 10^8^-10^5^ CFU of target strains. However, a few false-positive results were shown at 10^6^-10^5^ CFU. To validate this NGS panel analysis, three sets of qPCR analyses were independently performed with the same contaminated food samples, showing the similar specificity and selectivity for detection and identification. While this NGS panel still has some issues for detection and identification of specific foodborne pathogens, it has much more advantages, especially multiple detection and identification in one reaction, and it could be improved by further optimized NGS panel primer sets and even by application of a new real-time NGS sequencing technology. Therefore, this study suggests the efficiency and usability of NGS panel for rapid determination of origin strain in various foodborne outbreaks in one reaction.

## Introduction

Foodborne pathogens cause food poisoning, which is the most widespread health problem, thereby resulting in economic and health loss [1]. In particular, foodborne pathogens, including *Listeria monocytogenes*, *Salmonella* spp., and pathogenic *E. coli*, cause 746 foodborne outbreaks in the United States, of which 935 patients were hospitalized and 25 died (2020) [2]. In South Korea, the bacterial origin of foodborne outbreaks, including pathogenic *E. coli* (average of 39 cases) and *Salmonella* spp. (average of 18 cases), was reported in 2017 [3]. Thus, an efficient foodborne pathogen detection method must be developed.

Foodborne pathogen detection methods can be divided into (a) culture-based methods and (b) culture-independent methods. For culture-based methods, genus-specific selective media are used to isolate and count viable colonies of foodborne pathogens [4]. In addition, biochemical tests are coupled with culture-based methods to confirm the biological and chemical characteristics of specific foodborne pathogens [5]. However, culture-based methods using selective media do not always provide an accurate information of all species [6], and it requires up to three days of incubation [7]. Therefore, culture-based methods are time consuming and less accurate. Moreover, culture-independent methods are developed and divided into three types: (a) immunological methods (b) biosensors, and (c) nucleic acid–based detection. Immunological methods are quick, sensitive, and highly specific, which use antibodies such as enzyme-linked immune sorbent assay and immuno-magnetic separation [8]. However, these methods cause environmental stress on the antibody, thereby leading to low accuracy [9]. Electrical and optical biosensors are sensitive, easy to design, specific, and accurate [10, 11]. However, these technique needs additional instruments and compatible software, and it is not always cost-effective [12]. Recently, a DNA-based method has been used to detect foodborne pathogens in laboratory by specific gene-based polymerase chain reaction (PCR) [12]. The conventional PCR using a single primer set targeting a specific gene of foodborne pathogens has a high level of sensitivity and specificity [13]. However, conventional PCR requires a gel electrophoresis step to simultaneously confirm target gene PCR amplicon bands and the presence of a single-target gene [14, 15]. Therefore, real-time PCR or multiplex PCR has been developed to address these limitations of conventional PCR [16, 17]. Real-time PCR can detect specific foodborne pathogens by using a fluorescence signal from a probe using associated foodborne pathogen-specific gene-targeting primer sets [18]. Therefore, real-time PCR does not need an electrophoresis step. In addition, the determination of fluorescence intensity in real-time PCR enables quantification of DNA concentration and colony-forming unit (CFU) of foodborne pathogens [19]. The multiplex PCR can simultaneously detect several target genes using a mixture of primer sets [20]. Combining real-time PCR and multiplex PCR, multiplex real-time PCR was developed. Many recently developed multiplex real-time PCR for foodborne pathogen detection performed rapid and multiple detection based on high specificity and sensitivity [21, 22]. Recently, next-generation sequencing (NGS) was emerged to efficiently generate large quantities of DNA sequences and provide insights into genomic research [23]. The most used NGS sequencer is Illumina next-generation sequencers, which is the prevailing high-throughput technology, and it provides highly accurate sequencing outputs [24]. NGS produces a large number of DNA sequences at the same time, but this technology is expensive [25]. However, the cost of NGS service has been reduced and popularized because of the continuous development of new NGS technologies, including nanopore (Oxford Nanopore, U.K.) sequencing [26]. Given the cost reduction for NGS service, this NGS sequencing service is available for rapid detection and identification, microbial genomics, metagenomics, and meta-shotgun regarding the molecular studies of foodborne pathogens [27-29]. In particular, the NGS panel for the detection and identification of foodborne pathogens has been recently considered because it can screen several target gene sequences and efficiently analyze a large number of samples [30].

The NGS panel has been evaluated and used for cancer diagnosis and GMO detections. In a previous study, the NGS panel with 571 cervical cancer-candidate gene target primers was developed and evaluated using 32 tumors and 32 blood samples from Chinese patients with cervical cancer. NGS analysis revealed novel genetic alterations in Chinese patients with cervical cancer among 571 candidate genes, indicating the large screening diagnosis ability of the NGS panel [31]. In addition, the NGS panel successfully detected eight categories of GMO genes (maize endogen gene, *bt11* gene, *bt176* gene, soybean endogen gene, 35S/CTP4 construct, CP4-EPSPS element, p35S promoter, and tNOS terminator) in low amount of GM targets (>0.01%, of total sample), indicating the sensitive detection of the NGS panel [32]. However, the NGS panel was also applied for multiple detection and determination of various pathogens [33]. Previously, NGS, including 16S rRNA-based metagenome and random genome sequencing–based meta-shotgun approaches, was used to detect foodborne pathogens [34, 35]. However, this method has high detection limit, and it depends on the amount of the target foodborne pathogens present in the sample, generating low reads of target foodborne pathogens [36]. In addressing this disadvantage, the NGS panel was developed and evaluated using specific primer sets targeting foodborne pathogens [37]. At present, only one study on NGS panel for the detection and identification of foodborne pathogens was reported: species-specific multiplex PCR amplicon was sequenced by using an Illumina MiSeq sequencer, and it fully detected five to six foodborne pathogens (in 10^1^, 10^3^, and 10^5^ CFU per target foodborne pathogen) in food matrices [37]. This result indicates that the NGS panel approach can perform accurate species-specific identification *via* single NGS of only target-specific genes of foodborne pathogens, compared with metagenome and meta-shotgun sequencing [38]. However, only one primer set per one pathogen is used, and the specificity and sensitivity of primer sets are not fully evaluated, indicating the importance of the quality of NGS panel primer sets and requirement of multiple primer sets per one pathogen. Therefore, the NGS panel must be optimized with reliable multiple primer sets.

In this study, seven foodborne pathogens, namely, *Listeria monocytogenes*, *Salmonella enterica* serovar Typhimurium, and five pathogenic *E. coli* [enteropathogenic *E. coli* (EPEC), enteroinvasive *E. coli* (EIEC), enterotoxigenic *E. coli* (ETEC), enterohemorrhagic *E. coli* (EHEC), and enteroaggregative *E. coli* (EAEC)], were selected to optimize the NGS panel for the detection and identification of foodborne pathogens. In addition, one to three species-specific primer sets per single pathogen were designed and evaluated. Using these new primer sets, the NGS panel was validated using seven selected foodborne pathogens. In verifying the detection of the NGS panel, multiplex real-time PCR was performed as a control and compared with the NGS panel results. This study provides new insights into the importance of the NGS panel for the detection and identification of foodborne pathogens selected from the contaminated fermented food samples. Therefore, this technology would be helpful to food safety by preventing foodborne outbreaks via accurate detection and identification of pathogens in foods.

## Materials and Methods

### Bacterial Strains, Media, Growth Conditions, and Bacterial Isolation

The bacterial strains and media used in this study are described in Table 1. All bacterial strains were cultured at 37°C for 24 h. All media were purchased from BD (USA), and the agar medium was prepared with 1.8% (w/v) Bacto Agar (BD). Four samples (chicken breast and three animal byproducts) were collected (200 g for each sample) from the Garak market (Korea) to isolate the foodborne pathogens (Table 1). After sample collection, the collected samples were cut using a sterile scalpel. Twenty-five grams of collected samples was transferred into 3M sterilized bag (USA) and suspended with 225 ml of sterilized phosphate-buffered saline (PBS) for homogenization. Homogenization was performed using BagMixer 400 (Interscience, France) with speed of 4 m/s for 30 s. After homogenization, samples were serially diluted up to 10^−5^, spread onto the selective agar plate specific for each pathogen (Table 1), and incubated as previously described. After incubation, a single colony was collected and streaked on fresh culture medium agar plate (Table 1). The selected bacterium was identified using 16S rRNA gene sequencing. In particular, pathogenic *E. coli* was further identified using pathogen type-specific gene PCR. The identified bacterium was stored at −80°C in 20% (w/v) sterilized glycerol solution.

### Genomic and Total DNA Isolation

Genomic DNA of pathogens was extracted using the Genelix Bacterial Extraction kit (Sanigen, Korea), according to the manufacturer’s instructions. In addition, total bacterial DNA was extracted from (a) six different fermented food samples (three different types of Kimchi and three different types of yogurt) contaminated with seven selected foodborne pathogens or (b) six different fermented food samples free from contamination of selected pathogens for NGS panel analysis using the QIAamp DNA Stool Mini Kit (Qiagen, USA), according to the manufacturer’s standard protocol.

### Bacterial Identification

All PCRs were conducted using a T100 Touch Thermal Cycler (Bio-Rad, USA). In identifying isolated bacteria, 16S rRNA gene sequencing was performed as follows. The PCR mixture (final volume of 25 μl) contains 1 μl of DNA template (4 ng/μl), 0.5 μl of forward and reverse primers (20 μM; 27F/1492R) previously described by Chen *et al*. [39], and 12.5 μl of the BioFACT 2X Taq PCR Master Mix (BioFact). The final volume was adjusted with molecular water. The PCR condition was as follows: 1 cycle of 95°C for 3 min; 35 cycles of 95°C for 30 s, 60°C for 30 s, and 72°C for 1 min; and 1 cycle of 72°C for 5 min. After PCR amplification, purified 16S rRNA gene amplicons were obtained using the NICSRO™prep PCR Clean-up S & V Kit (Bionics, Korea) and sequenced at Bionics using the 3730xl DNA Analyzer (Thermo Fischer, USA), according to the manufacturer’s standard protocols.

### Pathogenic Identification of *E. coli* Using PCR

Pathogenic identification of *E. coli* was performed using PCR to identify pathogenic types of isolated *E. coli*. The PCR mixture (final volume of 25 μl) contains as same to 16S rRNA gene PCR mixture except for the primer set. Previously developed primer sets, including EAEC (MP2-aggR-F/R), EHEC (MP4-*stx1A*-F/R), EIEC (MP2-*invE*-F/R), EPEC (MP3-*bfpB*-F/R), and ETEC (MP2-LT-F/R), were used to identify pathogenic types of *E. coli* [40]. The PCR condition was as follows: 1 cycle of 95°C for 3 min; 35 cycles of 95°C for 30 s, 63°C for 30 s, and 72°C for 30 s; and 1 cycle of 72°C for 5 min. In verifying the PCR results, agarose gel electrophoresis was performed with 2.5% agarose gel and ethidium bromide (0.2 μg/ml) and each PCR amplicon size was confirmed in the gel using the 100-bp DNA ladder (Bioneer, Korea) after gel running at 135 V for 20 min.

### NGS Genome Sequencing

Bacterial genomic DNA of seven foodborne pathogens were used for the NGS sequencing library preparation template. For sequencing library preparation, the sequencing barcodes were added to the sequencing library preparation template using the TruSeq Nano DNA LT kit (Illumina, USA). Then, the sequencing library was sequenced using the Illumina MiSeq sequencer in accordance with the Illumina MiSeq 2 × 150-bp paired-end run protocol. After sequencing, raw reads were filtered using Trimmomatic program [41] with default parameters, and filtered reads were assembled using Unicycler program [42]. Then, assembled contigs of each foodborne pathogen were annotated using Prokka program [43]. Genomic sequences of seven foodborne pathogenic bacteria (Table 2) were deposited in GenBank with BioProject accession numbers PRJNA870224 (*E. coli* NCCP 14039) and PRJNA857825 (other pathogenic bacteria).

### Design and Optimization of NGS Panel Primer Sets

Publicly available complete genomic sequences of seven foodborne pathogens were collected from the GenBank database in NCBI (https://www.ncbi.nlm.nih.gov/genbank/) and VFDB [44]. Comparative pan-genomic analysis was performed using complete genomic sequences of target and other pathogens using ANVIO program to elucidate target pathogen-specific genes [45]. Among target pathogen-specific genes, virulence factors, toxin genes, and antibiotic-resistant genes were primarily considered for selection. After selecting target pathogen-specific genes, new primer sets for the NGS panel were designed with sequences of the selected genes using Primer3 program [46] using the following parameters: size of 100 to 300 bp, GC content of 40% to 60%, T_m_ of 53°C to 60°C, and self-compatibility of >4 [47]. After designing the primer set, the stability, such as self-binding and dimer formations, and specificity of the primer set to the genomic sequence was confirmed using Primer3 program. For NGS panel sequencing analysis, one to three genes per target pathogen were selected for primer design.

### Singleplex PCR

Singleplex and cross-check PCRs were performed to validate primer specificity to the genomic sequence of target pathogens. For singleplex PCR, the PCR mixture (final volume of 25 μl) contained 1 μl of DNA template (4 ng/μl), 0.5 μl of forward and reverse primers (20 μM), and 12.5 μl of the KAPA HiFi HotStart ReadyMix (Roche, Germany). The final volume was adjusted with molecular water. The PCR conditions for singleplex PCR were as follows: 1 cycle of 95°C for 3 min; 35 cycles of 95°C for 30 s, 60°C for 30 s, and 72°C for 30 s; and 1 cycle of 72°C for 5 min. The singleplex PCR results were verified in accordance with the previously reported method.

### Cross-Check PCR

For cross-check PCR, two approaches were performed as follows: (a) a single primer set with a genomic DNA mixture of seven target pathogens and (b) multiple primer sets (two to three primer sets per reaction) with single genomic DNA of target pathogen. The PCR mixture of the first cross-check PCR was prepared using the same composition as that of singleplex PCR except for DNA template. The test DNA template contained genomic DNA mixtures of a target pathogen and other non-target pathogens, whereas the negative control DNA template contained a mixture of genomic DNAs of non-target pathogens. These DNA templates contained 4 ng/μl of DNA per pathogen. In addition, the PCR mixture of the second cross-check PCR had similar composition to singleplex PCR except for multiple primer sets. The multiple primer sets contained two to three primer sets (20 μM each) per reaction with a single-target strain. The cross-check PCR was used under similar condition to that of the singleplex PCR, and PCR amplicons were checked using agarose gel electrophoresis as previously described.

### Multiplex PCR

Multiplex PCR was performed to confirm the specificity of the primer sets in the multi-detection of target pathogen-specific genes. The multiplex PCR mixture (final volume of 25 μl) contains 1 μl of DNA template (4 ng/μl per pathogen, a total of 19 pathogens), 0.5 μl of forward and reverse primer sets (2–3 primer sets, 20 μM each), and 12.5 μl of the KAPA HiFi HotStart ReadyMix (Roche). Moreover, the final volume was adjusted with molecular water. The test and negative control DNA templates were prepared using the same composition as that of the first cross-check PCR. The PCR results were verified in accordance with the previously described method.

### Fermented Food Sample Collection and Simulation with Selected Pathogens

Six fermented food samples (200 g, each sample), including three kimchi samples prepared with different types of vegetable (cabbage, radish, and leaf mustard), and three yogurt samples prepared in different forms (Greek, yogurt, and liquid yogurt), were collected from a market in Seoul, South Korea. After sample collection, 25 g of the six collected fermented food sample was separately transferred into a sterilized 50-ml conical tube (SPL, USA). In addition, for the preparation of the contaminated fermented food samples, seven pathogens were selected, including EAEC NCCP 14039, EHEC SG_006, EIEC SG_007, EPEC SG_010, ETEC SG_009, *L. monocytogenes* SG_004, and *Salmonella enterica* serovar Typhimurium SG_011 (Table 1). The seven selected pathogens were separately inoculated into sterilized LB culture media and incubated up to 1.0 optical density at a wavelength of 600 nm. Then, the CFU of each culture was adjusted to 1.0 × 10^8^ CFU/ml with sterilized LB broth medium. Each CFU-adjusted culture of a selected pathogen (1.0 × 10^8^ CFU/ml per pathogen) was mixed, and the mixture containing the six selected pathogenic species was centrifuged at 13,000 ×*g* for 10 min to harvest the bacterial cell mixture (1.0 × 10^8^ CFU per pathogen). This mixed cell pellet (7 × 10^8^ CFU) was resuspended with 1 ml of PBS (Difco). Then, the resuspended bacterial mixture was serially diluted 10-fold up to 7 × 10^5^ CFU per sample (1.0 × 10^5^ CFU per target pathogen in the sample). These serially diluted bacterial mixture (10^8^, 10^7^, 10^6^, and 10^5^ CFUs per target pathogen) was transferred to a 50 ml conical tube (SPL, Korea) containing 25 g of collected sample. After contamination, each of the six contaminated fermented food samples was transferred into a sterilized 3M bag containing 224 ml of PBS (Difco) and homogenized as previously described. The homogenized samples were used for total bacterial DNA extraction before NGS panel analysis. This experiment was performed in triplicate.

### NGS Panel Analysis

In preparing the NGS panel sequencing template by PCR, two types of DNA templates were prepared: (a) total DNA for test from one of seven different fermented food samples containing seven target pathogens and (b) total DNA for the negative control from one of seven fermented food samples without contamination of seven target pathogens. The PCR mixture (final volume of 25 μl) contains 1 μl of DNA template (total DNA for test or total DNA for negative control), 0.1 μl of forward and reverse primer per primer set (13 primer sets, 100 μM each), and 12.5 μl of the KAPA HiFi HotStart ReadyMix (Roche). The final volume was adjusted with molecular water. The PCR condition was similar to the singleplex PCR. After PCR, target PCR amplicons were purified by gel extraction using the NICSROprep DNA Gel Extraction S & V Kit (Bionics), according to the manufacturer’s protocol. Sequencing barcodes were added to the NGS panel sequencing template using the TruSeq Nano DNA LT kit (Illumina) to prepare the sequencing library for the NGS panel. After barcoding, the sequencing library was sequenced using Illumina MiniSeq, according to the Illumina MiniSeq 2 × 150-bp paired-end run protocol. After NGS sequencing, the raw reads were filtered by Trimmomatic program to obtain qualified reads over 20 of Phred quality score. After obtaining qualified reads, they were merged by Pandaseq [48] with default parameters. After merging qualified reads, they were mapped to the seven selected pathogen-specific gene sequences by BLASTN [49] with > 95% of nucleotide identity. Finally, the number of mapped reads was counted. Detection criteria are necessary to determine the false-positive detection of the NGS panel. Hence, NGS panel analysis with six different fermented food samples containing no selected pathogens was performed and compared with the NGS panel analysis result of six different fermented food samples containing the seven selected pathogens.

### Quantitative Real-Time PCR (qPCR)

In evaluating NGS panel analysis, qPCR was performed as the control detection method for the seven selected pathogens and compared with the NGS panel analysis results. qPCR was carried out using a CFX96 deep-well plate reader (Bio-Rad). The DNA template for qPCR was also similar to the NGS panel sequencing template previously described. The Genelix Multiplex Real-Time PCR kit (#G103, Sanigen) was used to detect *L. monocytogenes* and *Salmonella* spp. In addition, the Genelix Multiplex Real-Time PCR kit (#G105, Sanigen) was used to detect EHEC and ETEC, and the Genelix Multiplex Real-Time PCR kit (#G106, Sanigen) was used to detect EAEC, EIEC, and EPEC. qPCR was performed according to the manufacturer’s standard protocols, and the cycle threshold (Ct) was determined automatically using CFX Manager version 3.1 (Bio-Rad). All tests were performed in triplicate.

### Statistical Analysis

Prism graph pad version 7.0 (USA) and R version 4.1.2 (R Core Team, 2020) were used to perform all correlations and visualizations.

## Results

### Identification of Isolated Foodborne Pathogens

A total of 88 pathogenic bacteria were isolated from four samples (chicken breast and three animal byproducts). These pathogens included *E. coli* (67 strains), *Listeria monocytogenes* (five strains), *Listeria amylovorus* (one strain), *Salmonella enterica* serovar *Typhimurium* (seven strains), *Streptococcus alactolyticus* (one strain), *Enterococcus faecium* (five strains), and *Bacillus licheniformis* (two strains) identified in molecular level using 16S rRNA gene sequencing. For further identification of pathogenic types of *E. coli*, pathogen type-specific gene PCR showed the exact PCR amplicon size of *stx1A* (EHEC target gene), *invE* (EIEC target gene), *bfpB* (EPEC target gene), and *elt* (ETEC target gene) gene in four strains of isolated *E. coli* (Fig. S1). Among isolated and identified foodborne pathogens, seven strains, including EHEC SG_006, EIEC SG_007, EPEC SG_010, ETEC SG_009, *L. monocytogenes* SG_004, and *S*. Typhimurium SG_011, were selected as target pathogens. In addition, one EAEC NCCP 14039) was selected (Table 1). Based on previous reports, these foodborne pathogens caused foodborne outbreaks in areas where the fermented food samples were collected [50-52].

### General Genomic Features and Primer Set Design

Genomic sequence information of selected target pathogens is required to design the specific primer sets and confirm their binding sites in the genomes. Therefore, NGS genome sequencing was performed, and their draft genomic sequences were obtained from isolated EAEC, EHEC, EIEC, EPEC, EPEC, ETEC, *L. monocytogenes*, and *S. enterica*. The general genomic features of foodborne pathogens are summarized in Table 3. Based on the obtained genomic sequences, primer sets targeting the selected pathogen-specific genes were designed to meet the criteria of the primer design given in the Materials and Methods. The selected pathogen-specific genes and their targeting primer sets are listed in Table 3, and the primer target genes and primer binding locations are listed in Table S1.

### Singleplex PCR

**Fig. 1:**
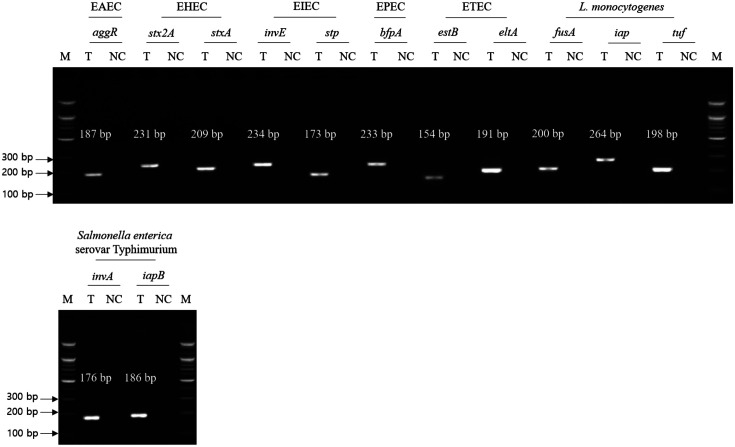
Result of singleplex PCR. Selected foodborne pathogens and their target genes were described. Test lane (T) contains the described target pathogen genomic DNA and specific gene primer set, and negative control lane (NC) contains molecular water and target pathogen-specific gene primer set. M: 100-bp DNA ladder.

Singleplex PCR was performed using a single genomic DNA of target pathogen and an associated single primer set to evaluate the specificity of primer sets to the selected foodborne pathogens. For the seven target pathogens, the selected specific genes with their genetic functions, designed specific primer sets, and predicted PCR amplicon sizes are described in Table 3. After singleplex PCR, gel electrophoresis analysis showed that all PCR amplicons had similar sizes to predicted PCR amplicon in single PCR bands, indicating that all PCR primer sets are specific to the associated target pathogens (Fig. 1). Therefore, these primer sets were confirmed with cross-check PCR evaluation.

### Cross-Check PCR

In evaluating the specificity of the primer, two different cross-check PCRs were conducted: (a) a single primer set with a genomic DNA mixture of the associated target pathogen and six different non-target pathogens; (b) a single selected pathogen-specific gene primer set (two to three primer sets) mixed with an associated target pathogen.

**Fig. 2:**
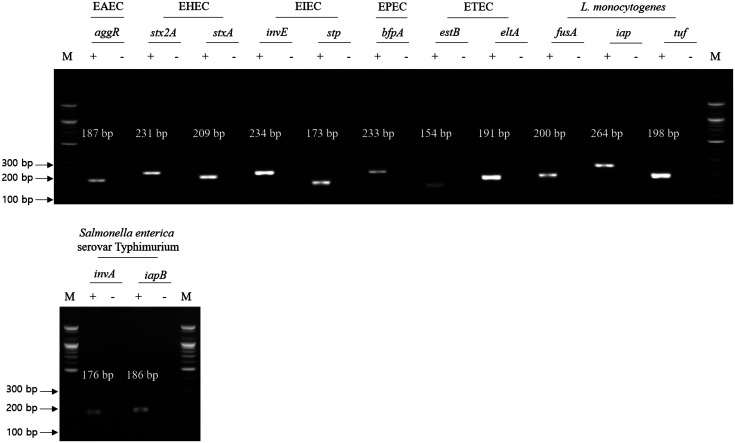
Result of first cross-check PCR. Selected foodborne pathogens and their target genes were described. Test lane (+) contains genomic DNA mixture, including target pathogen and associated single-target gene primer set, and negative test lane (−) contains genomic DNA mixture omitting target pathogen and associated target gene primer set. M: 100-bp DNA ladder.

For the first cross-check PCR, two types of genomic DNA templates were used: (a) test DNA template containing genomic DNA of target and non-target pathogens and (b) negative control DNA template containing only genomic DNA of non-target pathogens. Such templates were prepared to confirm the nonspecific binding of a single selected primer set to the genomic DNA of non-target pathogens. The gel electrophoresis result of the first cross-check PCR showed that the selected target gene-specific PCR amplicon bands were only observed in the test lanes, but no PCR amplicon bands were observed in the negative test lanes (Fig. 2). In addition, the sizes of PCR amplicon bands were similar to the expected ones, indicating that such primer sets are specific to the genomic DNA of target pathogens, although the DNA template contains all other genomic DNA of the non-target pathogens. Based on the first cross-check PCR results, primer sets are specific to the associated target gene and target pathogen.

**Fig. 3:**
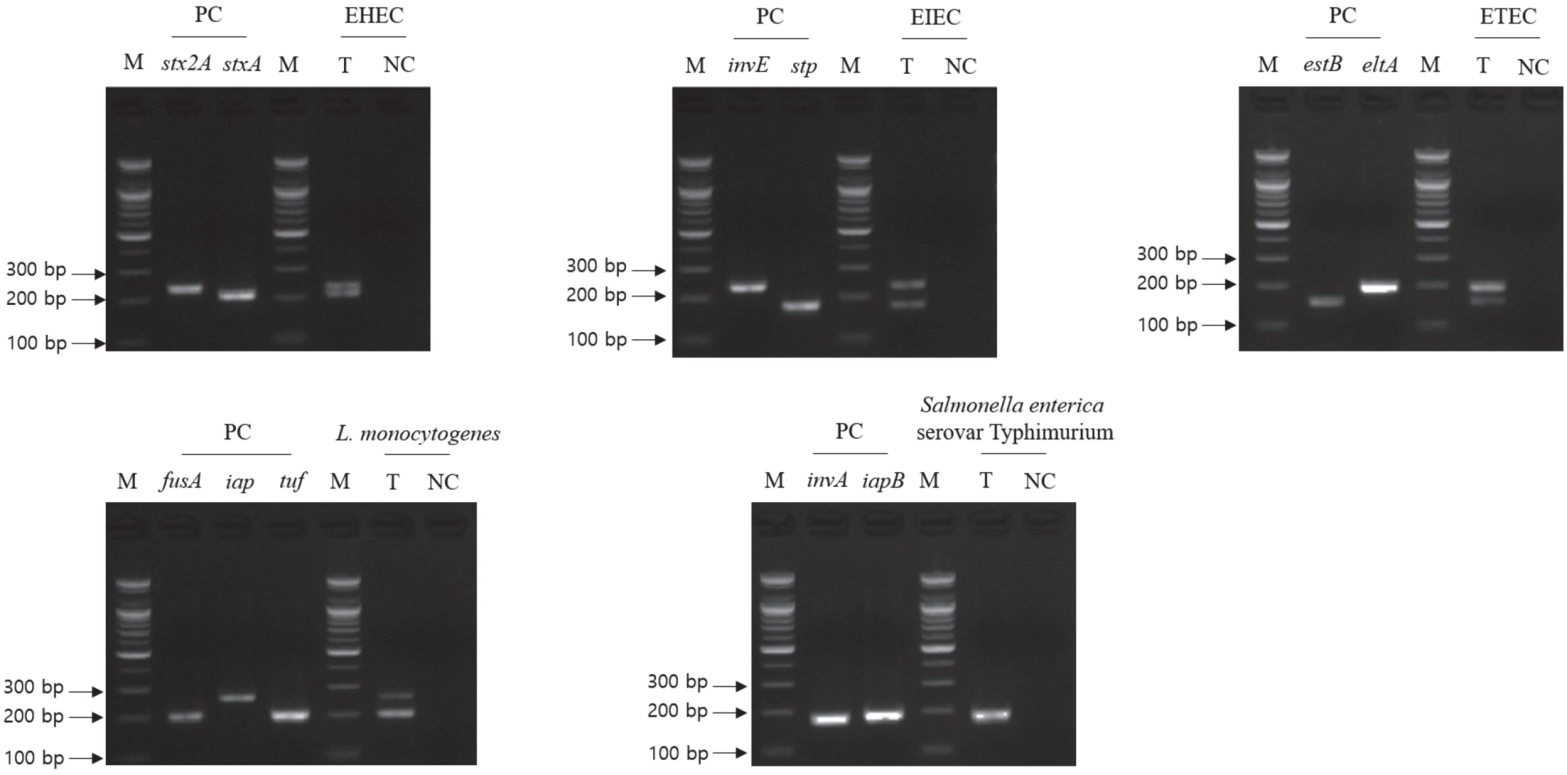
Result of second cross-check PCR. Selected foodborne pathogens and their target genes were described. Lanes labeled as target gene contain singleplex PCR amplicons of associated target gene for positive control (PC). Test lane (T) contains the described target pathogen genomic DNA and two to three target pathogen-specific gene primer sets, and negative control (NC) lane contains molecular water and two to three target pathogen-specific gene primer sets. M: 100-bp DNA ladder.

The second cross-check PCR was performed to evaluate whether one PCR (with multiple primer sets targeting single pathogen-specific genes) can multi-detect the target genes in a single pathogen. In particular, primer sets of EAEC and EPEC were omitted from the second cross-check PCR because only single-target gene was selected. Therefore, the second cross-check PCR primer set is a mixture of the primer sets targeting two to three selected genes in a single pathogen (a total of five combinations of primer mixture, Table 3). The gel electrophoresis result of the second cross-check PCR revealed that the PCR amplicons of all target genes in each pathogen were confirmed in the gel, and their amplicon sizes were similar to the expected ones (Fig. 3). Therefore, PCR with the mixture of primer sets targeting single-target pathogen-specific genes can detect target genes in one reaction without any primer interference.

### Multiplex PCR

Based on the results of the first cross-check and second cross-check PCRs, multiple target genes could be detected in one PCR, although primer sets and several pathogenic DNAs were mixed. Hence, multiplex PCR was performed with the mixture of primer set and several pathogenic DNAs. In particular, EAEC and EPEC targeting primer set mixtures were not tested in multiplex PCR because only single-target genes were selected.

**Fig. 4:**
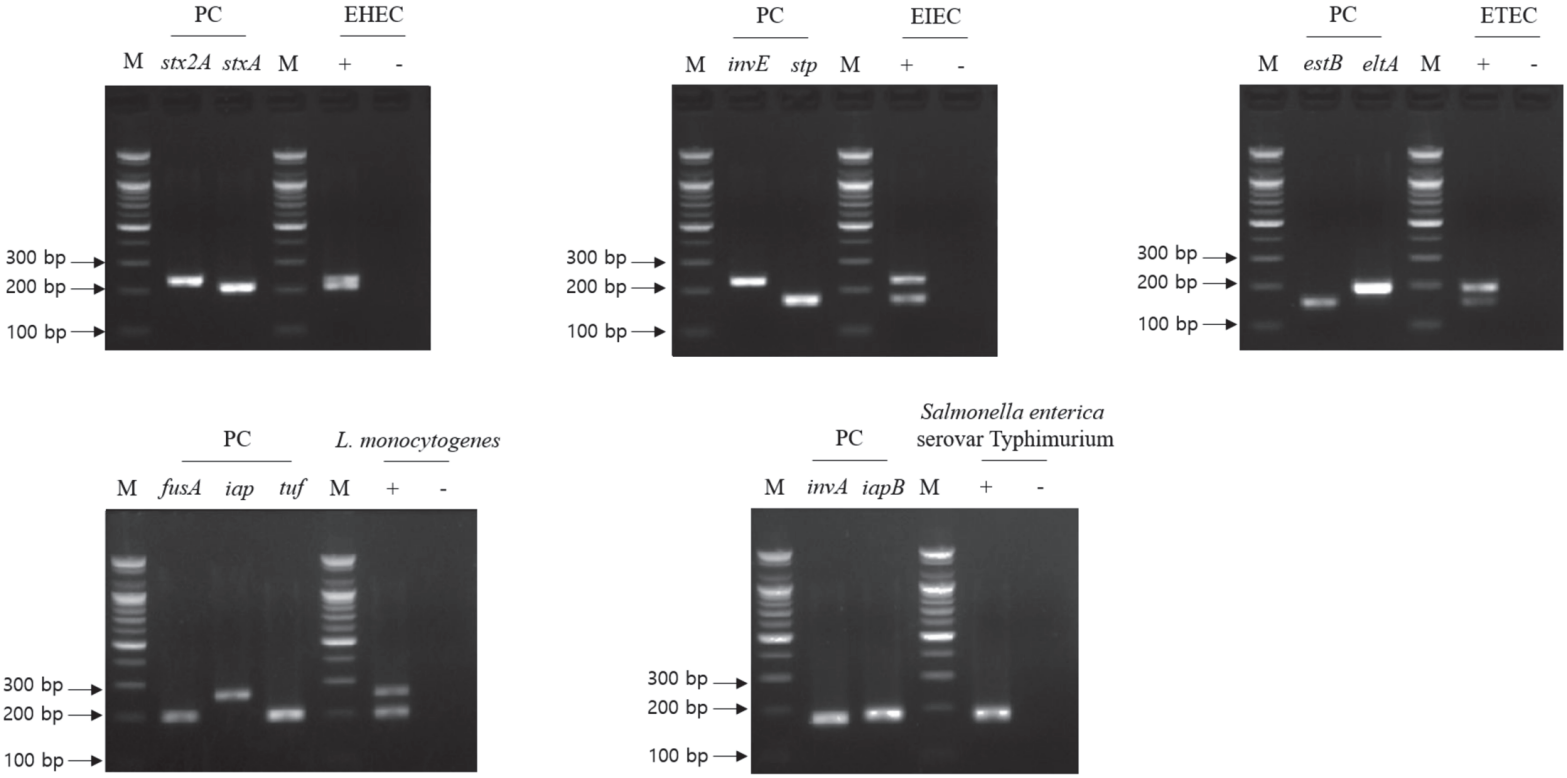
Result of multiplex PCR. Selected foodborne pathogens and their target genes were described. Lanes labeled as target gene contain singleplex PCR amplicons of associated target gene for positive control (PC). Test lane (+) contains genomic DNA mixture, including target pathogen and two to three target pathogen-specific gene primer sets, and negative test lane contains genomic DNA mixture omitting target pathogen and two to three target pathogen-specific gene primer sets. M: 100- bp DNA ladder.

For the multiplex PCR, DNA templates were prepared using the same procedure as that of the first cross-check PCR. In addition, the mixture of primer sets was prepared using the same mixture as that of the second cross-check PCR. The gel electrophoresis result of multiplex PCR showed that PCR amplicons of all multiple target genes per selected pathogen were detected in the gel, and their band sizes were the same to the expected ones (Fig. 4). Therefore, these primer sets will be susceptible for further NGS panel analysis.

### NGS Panel Analysis

**Fig. 5:**
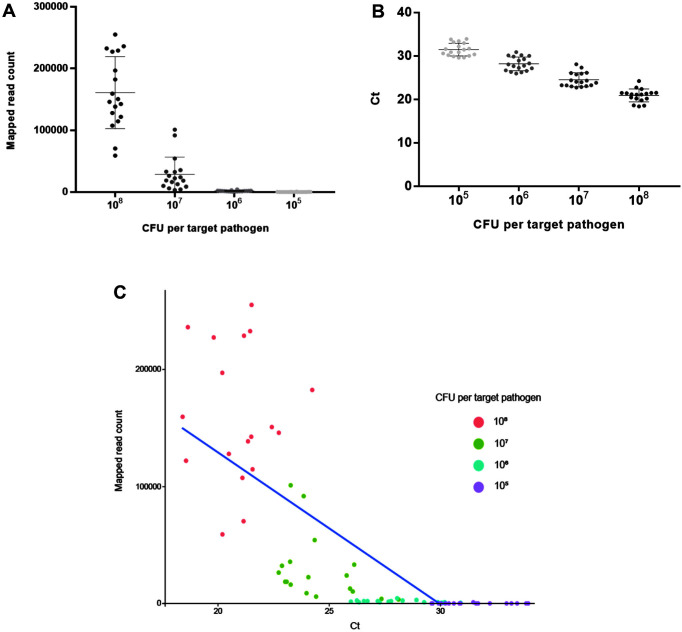
Dot plot of NGS panel analysis and qPCR result. Six different fermented food samples with 10^8^, 10^7^, 10^6^, and 10^5^ CFU per pathogen were plotted as dot using NGS panel analysis and qPCR. Each point is the (**A**) mean of six target pathogenspecific gene reads (NGS panel), (**B**) Ct values in a single replicate (qPCR), and (**C**) averages of mapped reads to total target pathogen-specific genes and averages of total target pathogen Ct values.

NGS panel analysis was performed with six different fermented food samples contaminated with the mixture of seven target pathogens. The NGS panel results showed that the average of the mapped sequence read to target pathogen-specific genes was obtained: 161,081 (54.77% of total qualified sequence reads), 28,929 (14.45%), 1,765 (1.23%), and 237 (0.15%) at 10^8^, 10^7^, 10^6^, and 10^5^ CFU per target pathogen, respectively (Table S2). In addition, the average of mapped sequence reads to target pathogen-specific genes and CFU per target pathogen was proportional (Fig. 5A). However, the prepared negative control without contamination in samples showed 1 to 3 mapped reads to target pathogen-specific genes in NGS panel analysis, indicating that a small number of those pathogens might be present in the original fermented food samples as a false-positive (Fig. S2A). Therefore, ≤3 reads were determined to be a false-positive for further NGS panel analysis.

**Fig. 6:**
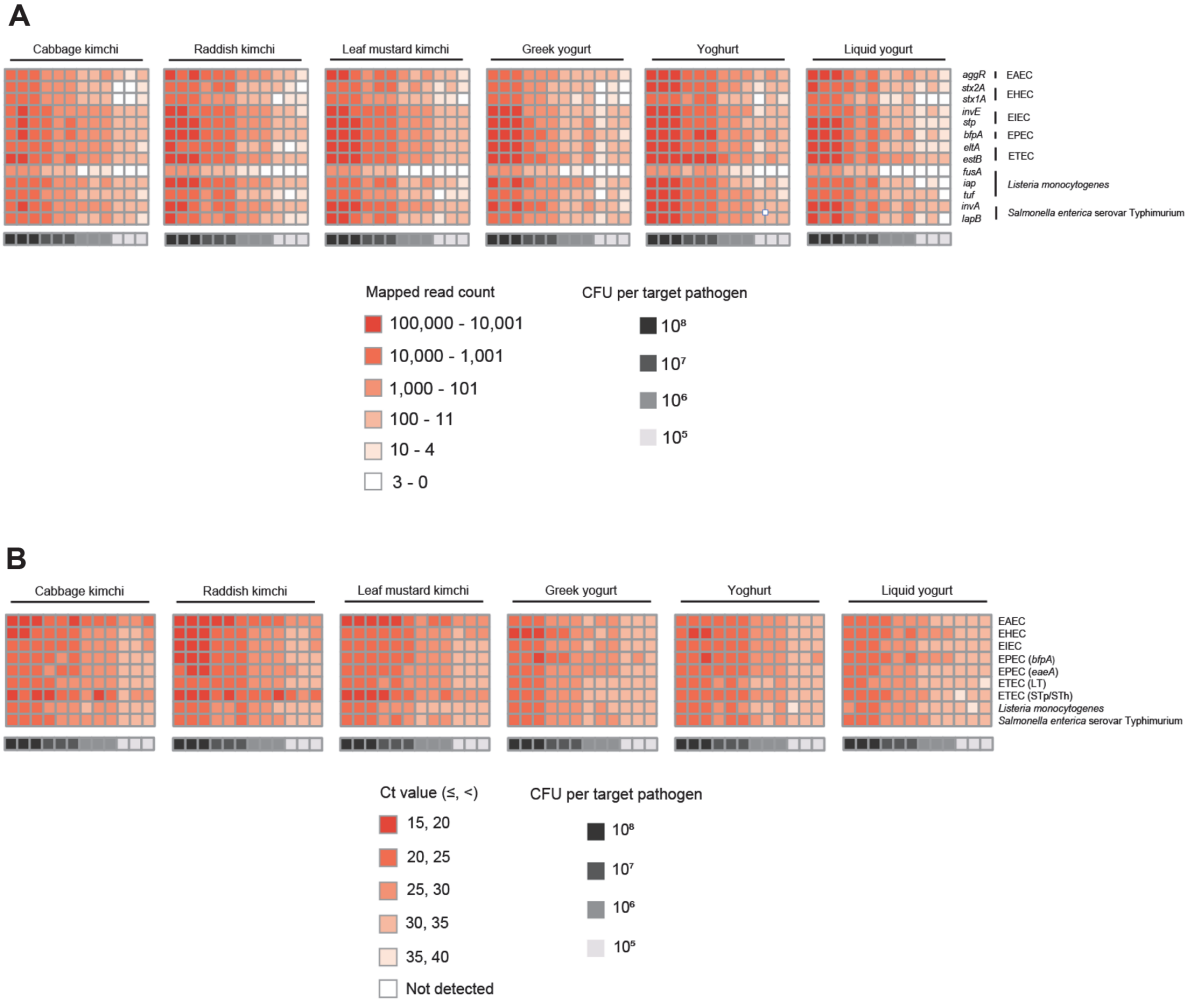
Target pathogen detection result in six different fermented food samples with or without a mixture of target pathogens. (**A**) Target pathogen-specific gene reads of the NGS panel; (**B**) target pathogen Ct values of qPCR were visualized using heat map. The color-scale of target pathogen-specific gene read or target pathogen Ct value and the level of CFU per target pathogen were indicated in the figure.

After mapping to 13 different target genes of seven target pathogens, all qualified NGS panel sequence reads were collected from six different fermented food samples. Then, the collected read counts in each dilution factor (10^8^, 10^7^, 10^6^, and 10^5^ CFU per target pathogen) were compared for the detection and identification of specific target pathogens (Fig. 6A). In dilution factors of 10^7^ to 10^8^, all 13 target genes multiplied, which was sufficient to identify seven different target pathogens in one NGS panel analysis without a false-positive (Figs. S2B and S2C). In addition, this result was confirmed in triplicate tests of all agricultural water samples. The serial dilution of target pathogens was proportionally associated with the read counts, showing the highest number of read counts in dilution factor of 10^8^ and lowest number of 10^7^, which is consistent with the result shown in Fig. 5A. However, in dilution factor of 10^6^, false-positive results were only detected in the *fusA* gene of *L. monocytogenes* (Fig. S2D). Furthermore, many false-positive reads appeared in dilution factor of 10^5^ (Fig. S2E). In particular, the *stxA* gene of EHEC and the *fusA* gene of *L. monocytogenes* were poorly detected by NGS panel analysis (Fig. S2E). Therefore, these two genes may be removed to increase the limits of detection and identify specific target pathogens in NGS panel analysis. Finally, this result indicates that the limits of detection and identification of target pathogens may be 10^7^ CFU.

### qPCR Analysis

qPCR was conducted to compare NGS panel analysis results with qPCR ones for evaluation. Two sets of qPCR DNA templates were prepared and used for qPCR, which were similar to those used for NGS panel analysis. qPCR was performed using three commercial qPCR detection kits, including all detection primer sets targeting seven different species of pathogens. Their qPCR results showed that the average Ct (threshold passed cycle) of target pathogens was 20.89 (10^8^ CFU per target pathogen), 24.51 (10^7^), 28.17 (10^6^), and 31.45 (10^5^). In addition, Ct and the cell number of target pathogens were negatively proportional, indicating that the rapid detection and identification of target pathogens were associated with low Ct or high cell number of target pathogens (Fig. 5B). However, the prepared negative control samples without specific pathogen contamination in samples showed no Ct during the whole qPCR (up to 40 cycles), thereby indicating the absence of target pathogens in negative control samples (Fig. S3A).

Furthermore, Ct values per target pathogen in four dilution factors (10^8^, 10^7^, 10^6^, and 10^5^ CFU per target pathogen) were compared to determine the sensitivity and detection limit by qPCR (Fig. 6B). In all dilution factors, all target pathogens were fully detected, and they clearly identified six different target pathogens in qPCR without a false-positive (Figs. S3B, S3C, S3D, and S3E). This result was confirmed in triplicate tests of all agricultural water samples. The highest cell number of target pathogens showed the lowest Ct values, which is consistent with the result shown in Fig. 5B. Therefore, the qPCR results indicate that the sensitivity of qPCR may be lower than 10^5^ CFU.

### Comparative Eevaluation and Correlation between the NGS Panel and qPCR

**Fig. 7:**
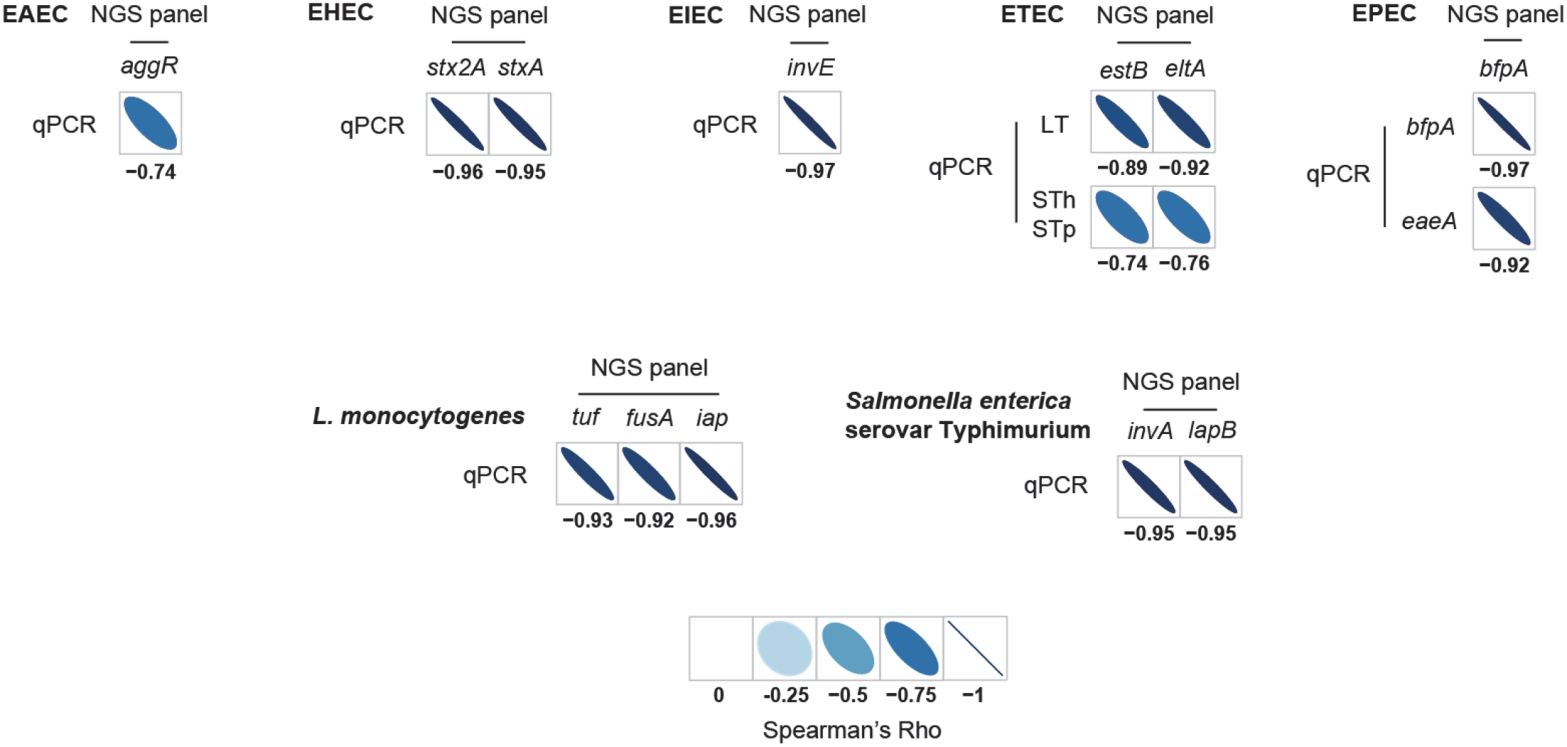
Spearman correlation analysis of selected foodborne pathogen detection results using the NGS panel and qPCR.

NGS panel analysis and qPCR results were compared to evaluate the ability of NGS panel analysis to detect and identify pathogens. Based on previous reports, the qualified read counts and Ct values were correlated with the cell number of target pathogens. Therefore, additional correlation analysis between the qualified read counts and Ct values in each specific target pathogen was performed. In addition, the read counts and Ct values were negatively correlated, and their comparative analysis showed a negative proportional relationship (Fig. 5C), indicating that the high cell number of target pathogens may be proportional to the rapid detection and identification of pathogens. Moreover, Spearman correlation analysis was performed to statistically compare the results between NGS panel analysis and qPCR in a specific target gene. This analysis revealed negative correlations (Fig. 7). Therefore, this high correlation between the NGS panel and qPCR indicates the importance of the newly developed NGS panel analysis for multiple detection and identification of target pathogens in foods.

## Discussion

Food consists of complex microbiota [53]. Thus, detecting and identifying the origin strain of a specific foodborne outbreak is difficult [54]. Several methods were developed and used to detect and identify outbreak-causing pathogens, such as culturing method using a selective medium, biochemical detection, and non-culturing methods, including an immunological method and PCR [55]. However, these methods may not be appropriate for the detection and identification of the origin of foodborne outbreaks in foods [56]. Therefore, the wide-screening method for the detection and identification of the foodborne outbreak origin must be developed and optimized.

Recently, the NGS panel was developed and performed in wide-screening range of genes for clinical cancer diagnosis and GMO detection and identification [57]. This method can also be used for multiple detection and identification of foodborne pathogens in food samples containing complex microbiota. Hence, in this study, NGS panel analysis was considered, developed, and comparatively evaluated using qPCR. The NGS panel primer sets targeting 13 pathogenic genes were developed and optimized. Using these primer sets, NGS panel analysis was performed by simulating seven pathogen-contaminated fermented food samples. The analysis revealed that all contaminated pathogens were fully detected and identified at 10^7^ CFU per pathogen in the samples, and they covered most target genes in the dilution factor of 10^6^ to 10^5^, indicating that this method can be used for multiple-pathogen detection in one reaction. In addition, subsequent detection and identification of specific pathogens in food samples using qPCR showed that these pathogens were fully detected and identified in the dilution factor of 10^8^–10^5^. However, comparative analysis of the NGS panel and qPCR showed that qPCR has higher sensitivity than the NGS panel, but all of the pathogens could not be detected in one reaction. Therefore, these two methods showed opposite advantage and disadvantage in multiple detection and sensitivity. Moreover, NGS panel analysis showed some false-positive results in low cell number of target pathogens. Based on these results, increasing the sensitivity of detection and identification by NGS panel analysis is necessary. Therefore, NGS panel analysis primer sets must be further optimized to enhance the sensitivity and avoid false-positive results. Furthermore, the NGS panel has longer analysis time than qPCR. Therefore, the application and optimization of a real-time NGS sequencing technology is necessary to reduce the NGS sequencing time. For example, nanopore sequencing is recognized as a real-time NGS sequencing [58]. Thus, the application of nanopore sequencing with NGS panel analysis may overcome the long sequencing time for NGS panel analysis.

Although NGS panel analysis described in this study still needs some improvements, this new foodborne pathogen detection method enables multi-detection and identification of various foodborne pathogens. Thus, this study provides new insights into the potential and advantages of NGS panel analysis to ensure food safety.

## Supplemental Materials

Supplementary data for this paper are available on-line only at http://jmb.or.kr.

## Figures and Tables

**Table 1:** Bacterial strains, culture medium, samples, and sampling locations.

Bacterium	Strain	Selective media^a^	Culture media^b^	References^c^	Sample	Sampling location
*Escherichia coli*						
EAEC	NCCP 14039	EMBA	LB	NCCP	-	-
EHEC	SG_006	EMBA	LB	This study	Chicken breast	Shin-won market, Seoul
EIEC	SG_007	EMBA	LB	This study	Pig intestine	Shin-won market, Seoul
EPEC	SG_010	EMBA	LB	This study	Pig intestine	Shin-won market, Seoul
ETEC	SG_009	EMBA	LB	This study	Chicken gizzard	Shin-won market, Seoul
*Listeria monocytogenes*	SG_004	OA	LB	This study	Cow intestine	Shin-won market, Seoul
*Salmonella enterica* serovar Typhimurium	SG_011	XA	LB	This study	Cow intestine	Shin-won market, Seoul

^a^OA: Oxford agar medium, XA: xylose–lysine–deoxycholate agar medium, EMBA: eosin–methylene–blue agar medium

^b^LB: Luria–Bertani medium

^c^NCCP: National Culture Collection for Pathogens

**Table 2:** General genome features of foodborne pathogens.

Bacterium	Strain	Genome size (bp)	Assembly	Contig	GC (%)	CDS	tRNA	rRNA	References
*Escherichia coli*									
EAEC	NCCP 14039^a^	4,966,374	Draft	105	50.61	4,828	80	4	This study
EHEC	SG_006	5,167,775	Draft	255	50.45	4,889	82	4	This study
EIEC	SG_007	4,927,911	Draft	385	50.78	4,667	51	2	This study
EPEC	SG_010	5,043,792	Draft	330	50.52	4,742	48	3	This study
ETEC	SG_009	5,030,956	Draft	201	50.28	4,794	85	3	This study
*Listeria monocytogenes*	SG_004	2,962,785	Draft	30	37.93	2,896	47	3	This study
*Salmonella enterica* serovar Typhimurium	SG_011	4,874,085	Draft	85	52.18	4,571	77	3	This study

^a^NCCP: National Culture Collection for Pathogens

**Table 3:** Selected pathogen species-specific genes, their functions, and primer sets.

Bacterium	Gene	Function	Primer^a^	Sequence (5′ to 3′)	Size (bp)	Reference
*Escherichia coli*						
EAEC	*aggR*	Transcriptional regulator	aggR_F	GATGCTGACGATTCTGTATTA	187	This study
			aggR_R	ATAAGTCCTTCTCGATTGTGT		
EHEC	*stx2A*	Shiga toxin 2 subunit A	stx2A_F	ACTGTCTGAAACTGCTCCTGT	231	This study
			stx2A _R	GGTTGACTCTCTTCATTCACG		
	*stxA*	Shiga toxin subunit A	stxA_F	GATAGATCCAGAGGAAGGGCG	209	This study
			stxA_R	TACGACTGATCCCTGCAACAC		
EIEC	*invE*	Invasion protein	invE_F	ACGAGTCAACTTTTAGCGAAGGG	234	This study
			invE_R	CTCTATTTCCAATCGCGTCAGAAC		
	*stp*	Type III secretion system	stp_F	TCCTGCTTAGATGATGGAGG	173	This study
		export apparatus protein	stp_R	CCAAAAGGAAGTGTCTGCTC		
EPEC	*bfpA*	Bundle-forming pilus	bfpA_F	TAGTGGATTGGACTCAACGAT	233	This study
		major subunit	bfpA_R	TATTAACACCGTAGCCTTTCG		
ETEC	*estB*	Heat-stable enterotoxin	estB_F	CTCAGGATGCTAAACCAGTAGAG	154	This study
		ST-I group b	estB_R	CCGGTACAAGCAGGATTACAAC		
	*eltA*	Heat-labile enterotoxin LT	eltA_F	TGACGGATATGTTTCCACTTC	191	This study
		subunit A	eltA_R	GTATTCCACCTAACGCAGAAA		
*Listeria monocytogenes*	*fusA*	GTP-binding protein	fusA_F	TTGATGGTGCTGTTGCGGTTC	200	This study
			fusA_R	TGGGAGTTGGATTGGGTGC		
	*iap*	Invasion-associated protein p60	iap_F	CTGGTGATACTCTTTGGGGTA	264	This study
			iap_R	AGCCGTTAGATTCGGTTGTTTC		
	*tuf*	EF-Tu/IF-2/RF-3 family GTPase	tuf_F	GTGACGAAGTAGAAGTTATCG	198	This study
			tuf_R	AGTTAGTGTGTGGAGTAATCG		
*Salmonella enterica* serovar	*invA*	Invasion protein	invA_F	CGCACTGAATATCGTACTGG	176	This study
			invA_R	CGATAATTTCACCGGCATCG		
Typhimurium						
	*iapB*	Lipopolysaccharide assembly protein	iapB_F	GCTGAGTAACCAACAAGATAA	186	This study
			iapB_R	AGTAAACGCTGTTCATAGGTC		

^a^F: Forward primer, R: Reverse primer. All primer sets were designed in this study
