# Supplementary material for: Development and Evaluation of a Next-Generation Sequencing Panel for the Multiple Detection and Identification of Pathogens in Fermented Foods
Source: J Microbiol Biotechnol. 2022 Nov 16;33(1):83–95. doi: 10.4014/jmb.2211.11009 (PMC9895999; doi:10.4014/jmb.2211.11009)
Supplement: Supplementary file 1 [file jmb-33-1-83-supple.pdf]

J. Microbiol. Biotechnol.
https://doi.org/10.4014/jmb.2211.11009

Supplementary Materials

Development and Evaluation of a Next-Generation Sequencing Panel for the Multiple Detection and Identification of Pathogens in Fermented Foods

Dong-Geun Park[1], Eun-Su Ha[2], Byungcheol Kang[2], Iseul Choi[2], Jeong-Eun Kwak[1], Jinho Choi[2], Jeongwoong Park[2], Woojung Lee[3], Seung Hwan Kim[3], Soon Han Kim[3] and Ju-Hoon Lee[1*]

[1] Department of Food and Animal Biotechnology, Department of Agricultural Biotechnology, Research Institute of Agriculture and Life Sciences, Center for Food and Bioconvergence, Seoul National University, Seoul 08826, Republic of Korea

[2] Research and Development Center, Sanigen Co., Ltd, Anyang 14059, Republic of Korea

[3] Division of Food Microbiology, National Institute of Food and Drug Safety Evaluation, Ministry of Food and Drug Safety, Cheongju 28159, Republic of Korea

***Corresponding author:**

Dr. Ju-Hoon Lee (juhlee@snu.ac.kr)

Table S1. *In silico* prediction of primer binding sites.

Bacterium	Strain	Gene	Contig	Primer binding site	
				Forward (nt)	Reverse (nt)
Selected food-borne pathogens					
Enteroaggregative *E. coli* (EAEC)	NCCP 14039	*aggR*	Contig 19	3,410-3,430	3,244-3,264
Enterohaemorhaggic *E. coli* (EHEC)	SG_006	*stx2A*	Contig 109	1,104-1,124	1,314-1,334
		stxA	Contig 93	452-472	640-660
Enteroinvasive *E. coli* (EIEC)	SG_007	*invE*	Contig 48	3,150-3,172	3,360-3,383
		stp	Contig 48	30,537-30,556	30,690-30,709
Enteropathogenic *E. coli* (EPEC)	SG_010	*bfpA*	Contig 48	18,505-18,525	18,717-18,737
Enterotoxigenic *E. coli* (ETEC)	SG_009	*estB*	Contig 239	-	153-174
		eltA	Contig 112	2,691-2,711	2,861-2,881
Listeria monocytogenes	SG_004	*fusA*	Contig 2	448,034-448,054	448,215-448,233
		iap	Contig 1	537,576-537,596	-
		tuf	Contig 2	450,653-450,673	450,830-450,850
Salmonella enterica	SG_011	*invA*	Contig 1	417,113-417,132	417,269-417,288
serovar *Typhimurium*		*iapB*	Contig 11	26,185-26,205	26,350-26,370

Table S2. Summary of NGS panel outputs.

Sample	Replicate	CFU	Yield (bp)	Raw read	Filtered read	Merged read	Mapped read to total target pathogen-specific gene
Cabbage Kimchi	1	10^8	53,429,124	355,190	339,952	165,794	70,542
	2		69,651,085	462,872	441,996	220,140	107,531
	3		45,070,533	299,620	286,467	141,013	59,030
	1	10^7	36,957,058	246,544	236,411	106,954	18,796
	2		31,106,357	207,564	198,680	93,804	6,017
	3		42,170,573	281,004	269,764	126,422	8,999
	1	10^6	35,832,348	238,836	228,900	104,828	1,284
	2		30,345,896	202,458	194,198	87,696	1,252
	3		34,723,694	231,976	222,001	94,260	2,392
	1	10^5	42,269,479	281,864	270,533	126,874	304
	2		33,933,097	226,258	215,239	101,478	212
	3		39,175,130	260,826	249,793	121,741	184
	1	N.C.	38,116,622	253,854	244,994	113,261	0
	2		33,716,443	224,394	216,563	108,260	0
	3		35,405,285	235,984	228,008	122,988	0
Raddish Kimchi	1	10^8	119,029,566	790,314	758,780	375,276	236,167
	2		92,341,378	613,376	588,105	284,809	159,487
	3		79,112,446	525,722	503,011	237,117	121,950
	1	10^7	64,101,041	426,938	408,025	179,054	18,574
	2		60,770,807	404,838	386,337	170,721	26,261
	3		63,346,114	422,010	402,598	173,925	32,547
	1	10^6	44,372,781	295,882	282,331	106,064	1,795
	2		56,277,277	375,088	358,322	129,738	1,877
	3		51,756,096	344,084	329,529	143,460	1,850
	1	10^5	42,264,437	281,760	268,996	116,120	175
	2		54,255,943	361,372	345,183	143,934	159
	3		36,070,553	240,524	229,628	91,684	123
	1	N.C.	45,089,840	301,420	290,931	118,670	1

	2		35,130,671	234,980	226,662	112,608	2
	3		37,241,290	249,118	240,200	143,976	1
Leaf mustard Kimchi	1	10^8	102,131,433	678,362	648,311	329,337	196,990
	2		114,155,954	758,706	725,247	366,860	227,464
	3		69,927,117	464,952	442,727	225,349	128,134
	1	10^7	57,899,344	384,348	369,551	183,601	16,458
	2		62,753,641	416,964	400,619	191,505	35,646
	3		49,583,195	329,482	315,545	154,093	22,435
	1	10^6	54,413,409	361,426	346,535	169,068	2,032
	2		47,695,192	316,630	303,300	149,370	1,553
	3		54,509,836	361,950	348,775	170,186	2,190
	1	10^5	53,041,047	352,716	340,371	162,866	228
	2		59,655,782	396,480	381,374	182,572	333
	3		60,192,138	400,126	384,641	188,017	181
	1	N.C.	40,838,316	272,172	261,966	137,520	1
	2		40,342,578	269,330	259,930	129,251	1
	3		42,880,990	286,462	276,722	130,875	7
Greek yogurt	1	10^8	79,557,479	527,854	504,998	247,209	142,687
	2		78,654,898	521,876	499,592	245,062	114,696
	3		85,604,438	567,800	541,170	272,147	138,466
	1	10^7	45,085,584	299,100	286,388	139,856	12,841
	2		45,038,325	298,690	284,233	142,209	10,128
	3		45,405,151	301,044	289,093	145,045	4,260
	1	10^6	50,406,799	334,258	321,289	161,604	780
	2		38,689,520	256,740	245,803	118,695	758
	3		42,624,682	282,578	269,353	135,503	1,148
	1	10^5	55,252,280	366,442	350,978	175,586	78
	2		40,496,261	268,656	255,599	129,743	326
	3		51,803,697	343,600	329,753	162,844	134
	1	N.C.	34,217,528	226,874	219,818	113,679	3
	2		37,755,625	250,352	242,366	123,292	0
	3		34,796,091	230,728	223,668	111,570	6

Yoghurt	1	10^8	123,533,914	819,892	787,588	401,774	255,198
	2		113,324,200	752,128	723,773	368,635	232,725
	3		108,881,103	722,692	693,206	353,341	229,070
	1	10^7	74,145,666	492,170	472,729	238,512	54,318
	2		86,573,630	574,806	552,963	278,651	101,121
	3		86,356,506	573,240	553,005	280,113	91,830
	1	10^6	57,250,201	380,058	363,335	184,915	4,418
	2		54,762,577	363,484	348,508	177,073	3,157
	3		60,084,440	398,596	380,619	194,364	2,371
	1	10^5	56,318,220	373,744	348,666	178,401	147
	2		58,463,476	388,228	373,786	189,204	991
	3		60,740,183	403,042	384,623	196,179	299
	1	N.C.	36,887,613	244,732	237,096	112,302	1
	2		39,327,825	260,890	252,150	127,852	1
	3		34,618,606	229,710	222,015	119,947	0
Liquid yogurt	1	10^8	97,580,132	647,754	619,965	315,706	182,400
	2		95,286,399	632,424	607,001	309,126	150,742
	3		90,083,525	597,908	572,975	291,904	146,181
	1	10^7	61,736,114	410,022	394,769	200,105	33,175
	2		50,732,505	336,658	322,687	164,636	3,311
	3		58,246,572	386,644	371,913	188,512	24,007
	1	10^6	51,357,594	340,764	327,406	166,586	1,080
	2		51,858,764	343,930	328,281	168,031	542
	3		53,581,987	355,512	340,438	173,389	1,296
	1	10^5	53,275,387	353,464	339,396	173,068	134
	2		59,637,304	395,996	380,513	193,341	193
	3		52,820,435	350,480	337,863	171,627	64
	1	N.C.	39,146,446	260,220	249,681	116,560	2
	2		38,573,027	256,254	245,107	123,915	2
	3		36,655,209	243,618	232,168	126,548	0

Supplementary Figures

Fig. S1. Gel electrophoresis result of pathogenic type-specific PCR of *E. coli*. Primer sets targeting each pathogenic type of *E. coli* were described above the Figure S1. Each lane contains single genomic DNA of isolated *E. coli* strains. Lane 1: EHEC SG_006, lane 2: EIEC SG_007, lane 3: EPEC SG_010, and lane 4: ETEC SG_009. M: 100 bp DNA ladder.

Fig. S2. Target pathogens-specific genes mapped read in six fermented food samples with target pathogens. Six different fermented food samples with (A) without target pathogens, (B) 10^8, (C) 10^7, (D) 10^6, and (E) 10^5 CFUs per target pathogen. Name of fermented food samples, number of replicates, target genes, associated target pathogens, and read color scales were indicated on the figure.

Fig. S3. Target pathogens Ct-value in six different fermented food samples with or without target pathogens. Six different fermented food samples with (A) without target pathogens, (B) 10^8, (C) 10^7, (D) 10^6, and (E) 10^5 CFUs per target pathogen. Name of fermented food samples, number of replicates, target genes, associated target pathogens, and read color scales were indicated on the figure. N.D. indicates Ct-values were not exceeded threshold until 40 cycles.

Fig. S1.

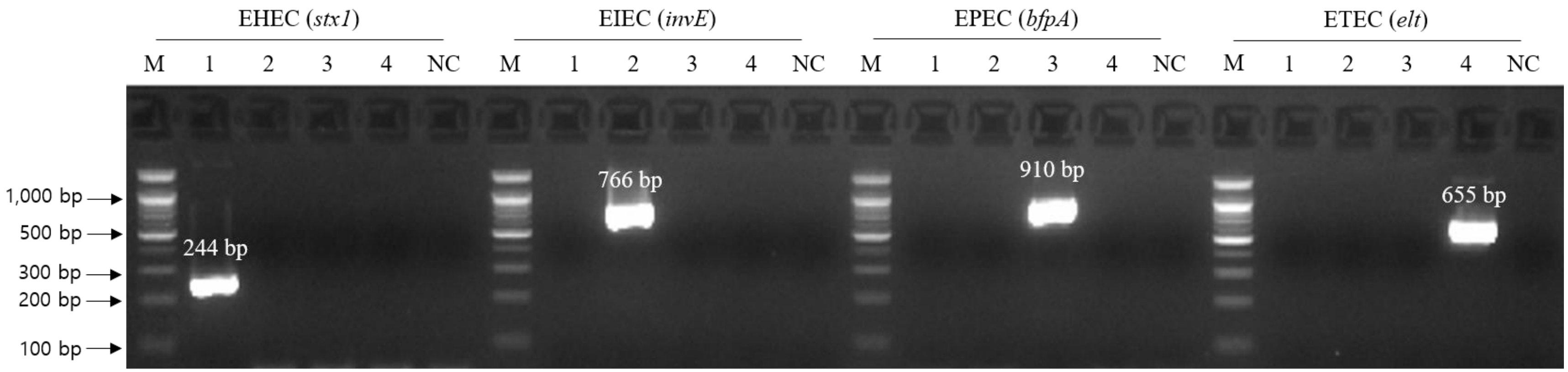
EHEC (*stx1*)
EIEC (*invE*)
EPEC (*bfpA*)
ETEC (*elt*)
M 1 2 3 4 NC M 1 2 3 4 NC M 1 2 3 4 NC M 1 2 3 4 NC
1,000 bp
500 bp
300 bp
200 bp
100 bp
244 bp
766 bp
910 bp
655 bp

Fig. S2.

A

Sample	Replicate	EAEC *aggR*	EHEC *stxA*	EHEC *stx2A*	EIEC *invE*	EIEC *stp*	EPEC *bfpA*	ETEC *eltA*	ETEC *estB*	*L. monocytogenes* *fusA*	*L. monocytogenes* *iap*	*L. monocytogenes* *tuf*	*S. enterica* serovar Typhimurium *invA*	*S. enterica* serovar Typhimurium *lapB*
Cabbage Kimchi	1	0	0	0	0	0	0	0	0	0	0	0	0	0
	2	0	0	0	0	0	0	0	0	0	0	0	0	0
	3	0	0	0	0	0	0	0	0	0	0	0	0	0
Raddish Kimchi	1	0	0	0	0	0	0	0	0	0	0	0	1	0
	2	0	0	0	0	0	2	0	0	0	0	0	0	0
	3	0	0	0	0	0	0	0	0	0	0	0	1	0
Leaf mustard Kimchi	1	0	0	0	0	0	1	0	0	0	0	0	0	0
	2	0	0	0	0	0	0	0	0	0	0	0	1	0
	3	0	1	0	0	2	2	2	0	0	0	0	0	0
Greek yogurt	1	0	1	0	0	0	2	0	0	0	0	0	0	0
	2	0	0	0	0	0	0	0	0	0	0	0	0	0
	3	0	1	0	0	0	3	2	0	0	0	0	0	0
Yoghurt	1	0	0	0	0	0	0	0	0	0	0	0	1	0
	2	0	0	0	0	0	0	1	0	0	0	0	0	0
	3	0	0	0	0	0	0	0	0	0	0	0	0	0
Liquid yogurt	1	0	0	0	0	1	1	0	0	0	0	0	0	0
	2	0	0	0	0	0	1	0	1	0	0	0	0	0
	3	0	0	0	0	0	0	0	0	0	0	0	0	0

Mapped read

- > 10,000
- 10,000 - 1,001
- 1,000 - 101
- 100 - 11
- 10 - 4
- 3 - 0

Fig. S2. Cont.

B

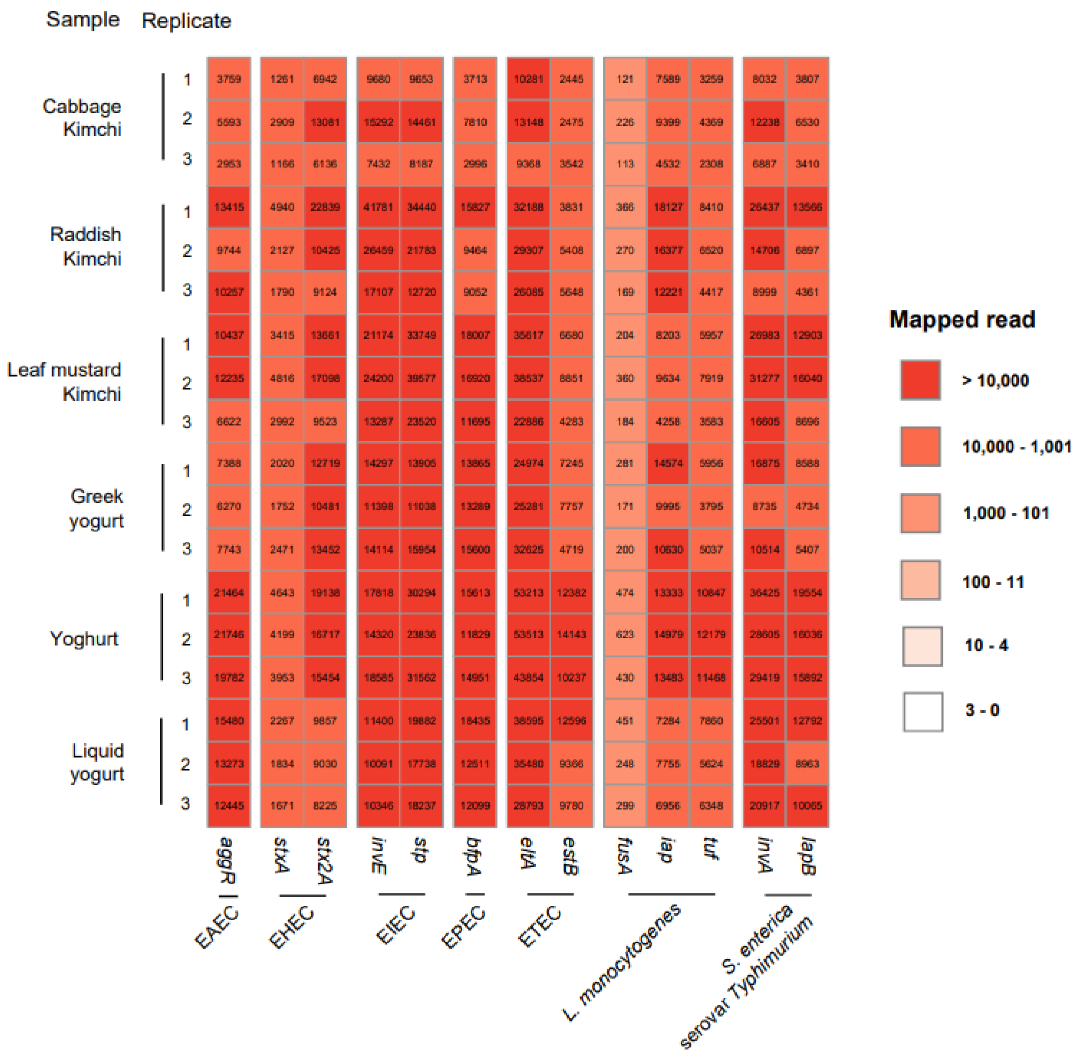
Sample
Replicate
Cabbage Kimchi
Raddish Kimchi
Leaf mustard Kimchi
Greek yogurt
Yoghurt
Liquid yogurt
1 3759 1261 6942 9680 9653 3713 10281 2445 121 7589 3259 8032 3807
2 5593 2909 13081 15292 14461 7810 13148 2475 226 9399 4369 12238 6530
3 2953 1166 6136 7432 8187 2996 9368 3542 113 4532 2308 6887 3410
1 13415 4940 22839 41781 34440 15827 32188 3831 366 18127 8410 26437 13566
2 9744 2127 10425 26459 21783 9464 29307 5408 270 16377 6520 14706 6897
3 10257 1790 9124 17107 12720 9052 26085 5648 169 12221 4417 8999 4361
1 10437 3415 13661 21174 33749 18007 35617 6680 204 8203 5957 26983 12903
2 12235 4816 17098 24200 39577 16920 38537 8851 360 9634 7919 31277 16040
3 6622 2992 9523 13287 23520 11695 22886 4283 184 4258 3583 16605 8696
1 7388 2020 12719 14297 13905 13865 24974 7245 281 14574 5956 16875 8588
2 6270 1752 10481 11398 11038 13289 25281 7757 171 9995 3795 8735 4734
3 7743 2471 13452 14114 15954 15600 32625 4719 200 10630 5037 10514 5407
1 21464 4643 19138 17818 30294 15613 53213 12382 474 13333 10847 36425 19554
2 21746 4199 16717 14320 23836 11829 53513 14143 623 14979 12179 28605 16036
3 19782 3953 15454 18585 31562 14951 43854 10237 430 13483 11468 29419 15892
1 15480 2267 9857 11400 19882 18435 38595 12596 451 7284 7860 25501 12792
2 13273 1834 9030 10091 17738 12511 35480 9366 248 7755 5624 18829 8963
3 12445 1671 8225 10346 18237 12099 28793 9780 299 6956 6348 20917 10065
aggR
stxA
stx2A
invE
stp
bfpA
eltA
estB
fusA
iap
tuf
invA
lapB
EAEC
EHEC
EIEC
EPEC
ETEC
L. monocytogenes
S. enterica serovar Typhimurium
Mapped read
> 10,000
10,000 - 1,001
1,000 - 101
100 - 11
10 - 4
3 - 0

Fig. S2. Cont.

C

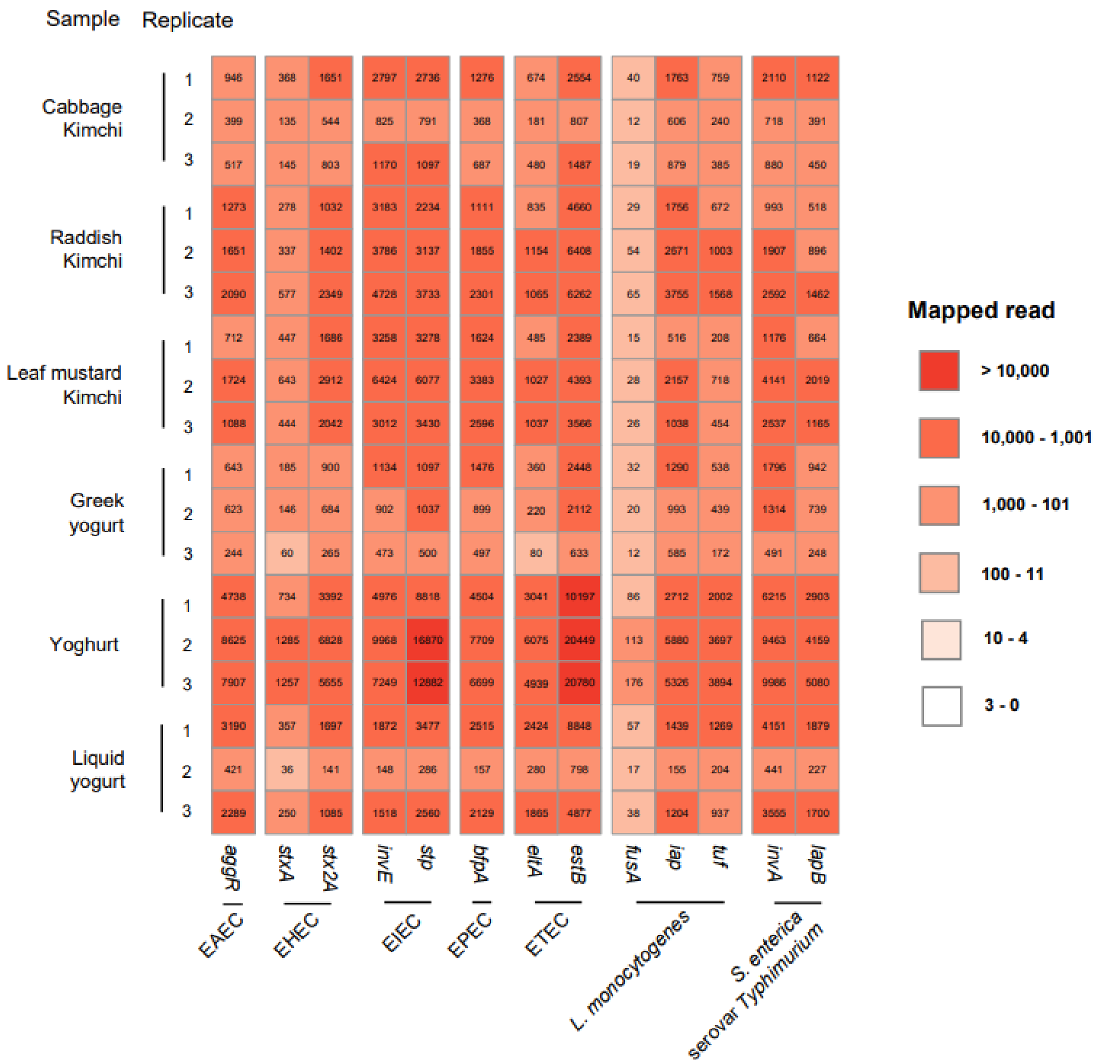
Sample
Replicate
Cabbage Kimchi
Raddish Kimchi
Leaf mustard Kimchi
Greek yogurt
Yoghurt
Liquid yogurt
aggR
stxA
stx2A
invE
stp
bfpA
eltA
estB
fusA
iap
tuf
invA
lapB
EAEC
EHEC
EIEC
EPEC
ETEC
L. monocytogenes
S. enterica serovar Typhimurium
Mapped read
> 10,000
10,000 - 1,001
1,000 - 101
100 - 11
10 - 4
3 - 0

Fig S2. Cont.

D

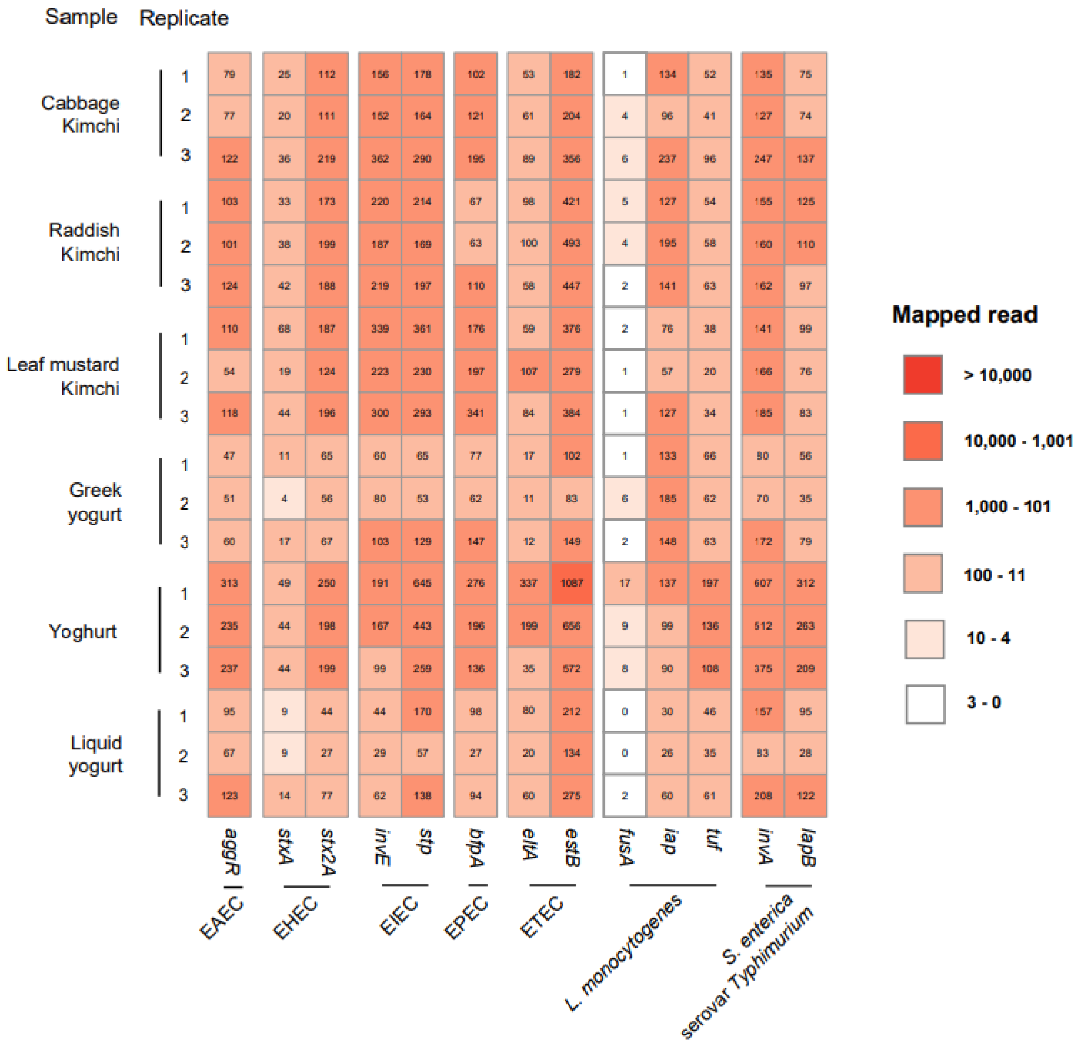

Sample
Replicate
Cabbage Kimchi
Raddish Kimchi
Leaf mustard Kimchi
Greek yogurt
Yoghurt
Liquid yogurt
1 79 25 112 156 178 102 53 182 1 134 52 135 75
2 77 20 111 152 164 121 61 204 4 96 41 127 74
3 122 36 219 362 290 195 89 356 6 237 96 247 137
1 103 33 173 220 214 67 98 421 5 127 54 155 125
2 101 38 199 187 169 63 100 493 4 195 58 160 110
3 124 42 188 219 197 110 58 447 2 141 63 162 97
1 110 68 187 339 361 176 59 376 2 76 38 141 99
2 54 19 124 223 230 197 107 279 1 57 20 166 76
3 118 44 196 300 293 341 84 384 1 127 34 185 83
1 47 11 65 60 65 77 17 102 1 133 66 80 56
2 51 4 56 80 53 62 11 83 6 185 62 70 35
3 60 17 67 103 129 147 12 149 2 148 63 172 79
1 313 49 250 191 645 276 337 1087 17 137 197 607 312
2 235 44 198 167 443 196 199 656 9 99 136 512 263
3 237 44 199 99 259 136 35 572 8 90 108 375 209
1 95 9 44 44 170 98 80 212 0 30 46 157 95
2 67 9 27 29 57 27 20 134 0 26 35 83 28
3 123 14 77 62 138 94 60 275 2 60 61 208 122
aggR
stxA
stx2A
invE
stp
bfpA
eltA
estB
fusA
iap
tuf
invA
lapB
EAEC
EHEC
EIEC
EPEC
ETEC
L. monocytogenes
S. enterica serovar Typhimurium
Mapped read
> 10,000
10,000 - 1,001
1,000 - 101
100 - 11
10 - 4
3 - 0

Fig. S2. Cont.

E

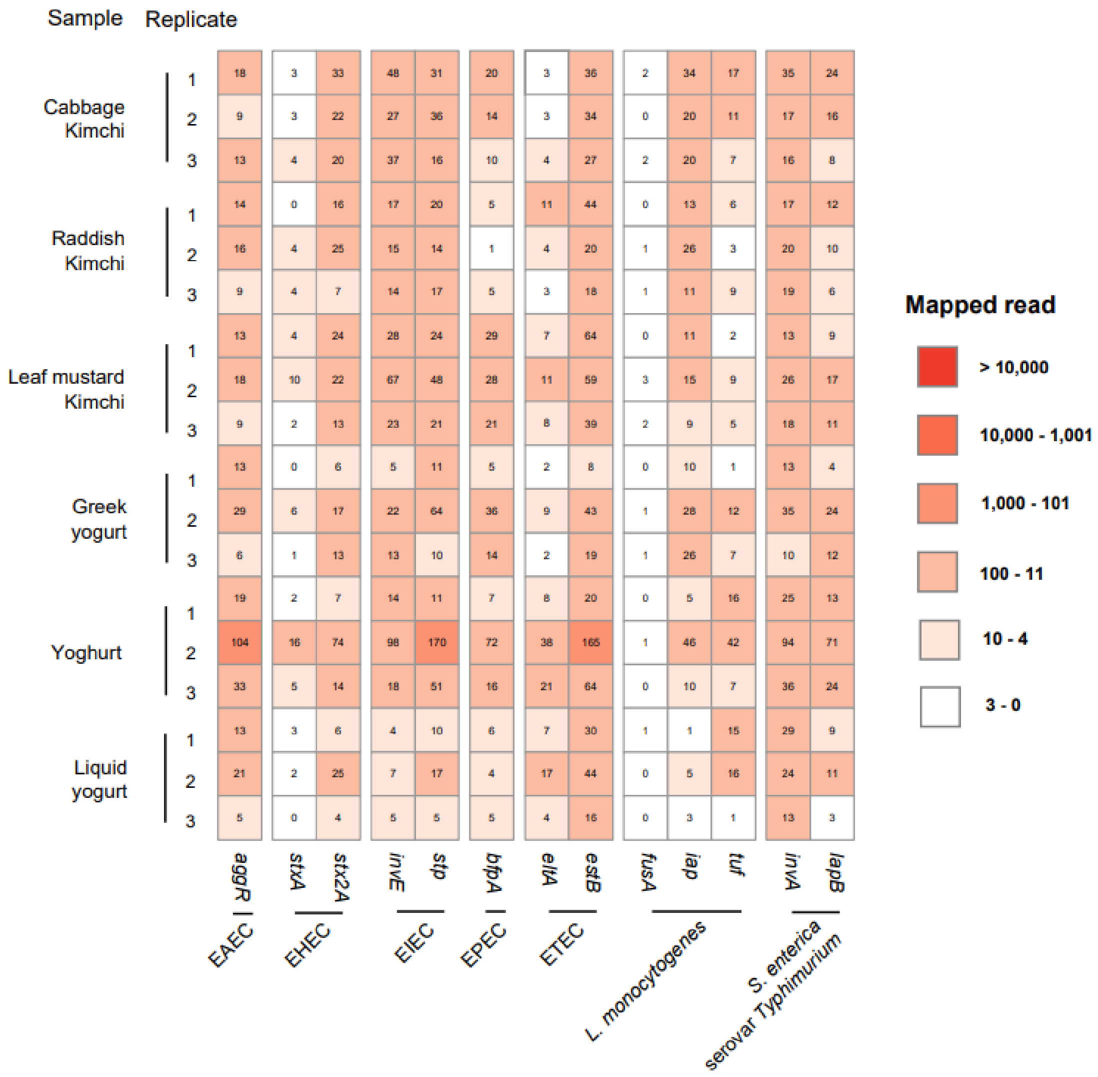

Sample
Replicate
Cabbage Kimchi
Raddish Kimchi
Leaf mustard Kimchi
Greek yogurt
Yoghurt
Liquid yogurt
aggR
stxA
stx2A
invE
sip
bfpA
eltA
estB
fusA
iap
tuf
invA
lapB
EAEC
EHEC
EIEC
EPEC
ETEC
L. monocytogenes
S. enterica serovar Typhimurium
Mapped read
> 10,000
10,000 - 1,001
1,000 - 101
100 - 11
10 - 4
3 - 0

Fig. S3.

A

Sample	Replicate	EAEC	EHEC	EIEC	EPEC (*bfpA*)	EPEC (*eaeA*)	ETEC (LT)	ETEC (STh/STp)	*L. monocytogenes*	*S. enterica* serovar *Typhimurium*
Cabbage Kimchi	1	N.D.	N.D.	N.D.	N.D.	N.D.	N.D.	N.D.	N.D.	N.D.
	2	N.D.	N.D.	N.D.	N.D.	N.D.	N.D.	N.D.	N.D.	N.D.
	3	N.D.	N.D.	N.D.	N.D.	N.D.	N.D.	N.D.	N.D.	N.D.
Raddish Kimchi	1	N.D.	N.D.	N.D.	N.D.	N.D.	N.D.	N.D.	N.D.	N.D.
	2	N.D.	N.D.	N.D.	N.D.	N.D.	N.D.	N.D.	N.D.	N.D.
	3	N.D.	N.D.	N.D.	N.D.	N.D.	N.D.	N.D.	N.D.	N.D.
Leaf mustard Kimchi	1	N.D.	N.D.	N.D.	N.D.	N.D.	N.D.	N.D.	N.D.	N.D.
	2	N.D.	N.D.	N.D.	N.D.	N.D.	N.D.	N.D.	N.D.	N.D.
	3	N.D.	N.D.	N.D.	N.D.	N.D.	N.D.	N.D.	N.D.	N.D.
Greek yogurt	1	N.D.	N.D.	N.D.	N.D.	N.D.	N.D.	N.D.	N.D.	N.D.
	2	N.D.	N.D.	N.D.	N.D.	N.D.	N.D.	N.D.	N.D.	N.D.
	3	N.D.	N.D.	N.D.	N.D.	N.D.	N.D.	N.D.	N.D.	N.D.
Yoghurt	1	N.D.	N.D.	N.D.	N.D.	N.D.	N.D.	N.D.	N.D.	N.D.
	2	N.D.	N.D.	N.D.	N.D.	N.D.	N.D.	N.D.	N.D.	N.D.
	3	N.D.	N.D.	N.D.	N.D.	N.D.	N.D.	N.D.	N.D.	N.D.
Liquid yogurt	1	N.D.	N.D.	N.D.	N.D.	N.D.	N.D.	N.D.	N.D.	N.D.
	2	N.D.	N.D.	N.D.	N.D.	N.D.	N.D.	N.D.	N.D.	N.D.
	3	N.D.	N.D.	N.D.	N.D.	N.D.	N.D.	N.D.	N.D.	N.D.

C_T value
- 15 >
- 15 ~ 20
- 20 ~ 30
- 30 ~ 35
- 35 ~ 40
- N.D.

Fig. S3. Cont.

B

Sample	Replicate	EAEC	EHEC	EIEC	EPEC (*bfpA*)	EPEC (*eaeA*)	ETEC (LT)	ETEC (STh/STp)	*L. monocytogenes*	*S. enterica* serovar *Typhimurium*
Cabbage Kimchi	1	18.35	19.78	20.45	22.72	21.05	22.17	19.30	24.05	22.28
	2	18.00	19.89	20.95	20.92	20.35	22.14	21.82	23.24	22.34
	3	16.35	20.33	21.09	21.60	21.21	22.76	11.64	23.24	23.36
Raddish Kimchi	1	13.43	18.84	19.20	19.96	20.18	20.60	11.26	23.08	21.07
	2	14.30	19.21	19.21	19.50	19.12	20.13	11.06	21.68	21.24
	3	13.34	19.14	19.98	19.61	19.05	20.62	11.20	22.10	21.82
Leaf mustard Kimchi	1	16.50	20.07	20.48	20.50	21.15	22.01	15.28	23.35	22.19
	2	19.15	20.57	20.60	20.35	20.87	22.12	7.86	24.24	22.30
	3	17.83	21.18	20.18	20.01	20.69	21.72	16.97	23.36	22.23
Greek yogurt	1	21.70	19.82	21.72	20.16	21.49	22.74	21.34	22.35	21.92
	2	21.24	19.24	21.40	20.07	21.22	22.48	22.08	23.59	22.46
	3	21.01	18.90	21.04	19.42	20.51	23.02	21.22	24.37	22.46
Yoghurt	1	21.26	20.20	21.58	20.34	21.17	22.89	21.28	23.00	21.66
	2	21.15	19.95	21.62	20.21	21.41	22.89	21.13	22.61	21.83
	3	21.01	19.72	21.05	19.09	21.03	23.03	21.28	22.61	21.49
Liquid yogurt	1	24.21	22.89	24.72	22.61	24.66	24.06	23.76	26.12	24.89
	2	22.11	21.25	22.72	20.79	22.59	23.39	21.71	24.02	23.01
	3	22.24	21.24	22.75	20.50	22.32	25.14	23.14	24.33	22.73

CT value

- 15 >
- 15 ~ 20
- 20 ~ 30
- 30 ~ 35
- 35 ~ 40
- N.D.

Fig. S3. Cont.

C

Sample	Replicate	EAEC	EHEC	EIEC	EPEC (*bfpA*)	EPEC (*eaeA*)	ETEC (LT)	ETEC (STh/STp)	*L. monocytogenes*	*S. enterica* serovar *Typhimurium*
Cabbage Kimchi	1	22.00	23.41	22.83	23.05	25.03	24.39	16.14	25.16	24.97
	2	20.76	22.27	24.73	25.17	24.71	26.24	21.57	27.21	26.80
	3	19.52	23.25	24.61	24.80	22.93	25.89	21.15	26.69	26.77
Raddish Kimchi	1	17.44	23.52	23.46	23.87	22.67	24.26	20.02	25.82	26.52
	2	19.37	23.13	22.93	22.57	23.01	23.38	19.29	25.55	25.15
	3	21.12	22.12	22.49	22.51	24.27	23.53	20.41	24.56	24.69
Leaf mustard Kimchi	1	17.15	21.81	23.91	24.23	23.05	25.78	18.49	28.26	26.51
	2	19.58	23.67	22.48	22.51	24.19	24.31	21.28	26.24	24.69
	3	21.74	24.38	23.77	23.26	25.17	25.14	20.37	26.71	25.80
Greek yogurt	1	26.12	23.67	25.94	24.29	25.44	29.39	25.44	27.12	25.81
	2	25.58	24.38	25.72	24.29	25.57	27.24	26.61	28.48	26.46
	3	27.48	25.95	27.17	25.67	27.49	27.27	27.87	29.00	28.02
Yoghurt	1	24.14	22.70	24.32	22.68	24.35	25.06	24.35	26.12	25.18
	2	22.86	21.58	23.05	22.25	23.15	24.40	22.94	24.83	24.15
	3	23.29	22.09	23.61	22.64	23.74	26.23	23.03	25.23	24.63
Liquid yogurt	1	24.96	24.26	25.78	23.62	25.91	27.22	24.54	29.59	29.03
	2	27.90	27.36	29.12	27.65	29.16	29.97	28.23	27.30	26.16
	3	25.32	24.84	26.09	24.03	26.19	26.46	26.00	27.04	25.89

CT value

- 15 >
- 15 ~ 20
- 20 ~ 30
- 30 ~ 35
- 35 ~ 40
- N.D.

Fig. S3. Cont.

D

Sample	Replicate	EAEC	EHEC	EIEC	EPEC (*bfpA*)	EPEC (*eaeA*)	ETEC (LT)	ETEC (STh/STp)	*L. monocytogenes*	*S. enterica* serovar *Typhimurium*
Cabbage Kimchi	1	23.32	26.49	27.53	27.41	26.14	28.77	25.59	30.12	29.82
	2	23.40	27.24	27.24	27.47	27.52	28.23	16.84	29.31	29.57
	3	23.73	25.97	25.94	25.68	27.82	27.50	23.39	28.06	27.93
Raddish Kimchi	1	24.06	25.05	26.00	26.63	26.44	26.61	21.90	28.63	28.24
	2	23.60	26.12	26.53	27.89	26.68	26.98	24.52	29.18	28.77
	3	23.15	26.55	27.14	27.71	27.64	27.47	19.29	30.25	29.66
Leaf mustard Kimchi	1	25.14	27.54	26.86	26.97	28.07	28.45	25.62	30.96	30.19
	2	24.56	26.22	27.50	27.29	28.12	29.12	25.34	30.99	29.72
	3	24.01	25.70	27.42	26.86	27.85	28.67	23.16	31.06	29.71
Greek yogurt	1	30.28	29.10	30.14	28.43	29.96	31.15	30.23	30.61	30.33
	2	29.97	30.08	29.68	28.15	29.59	32.87	31.26	29.95	29.49
	3	29.30	27.05	29.32	27.38	28.86	31.75	30.11	30.46	29.01
Yoghurt	1	27.69	26.53	27.74	26.15	27.76	30.34	27.39	30.02	28.70
	2	28.17	26.79	28.43	26.42	28.10	30.08	28.36	33.71	30.04
	3	28.28	26.70	29.21	26.97	28.63	29.33	27.89	29.04	28.46
Liquid yogurt	1	29.62	28.71	30.14	28.22	30.24	31.60	29.66	31.01	29.56
	2	30.25	28.80	30.86	29.65	31.23	31.86	32.01	32.86	30.46
	3	30.02	29.26	30.46	28.24	30.88	31.14	30.11	31.28	30.15

C_T value

- 15 >
- 15 ~ 20
- 20 ~ 30
- 30 ~ 35
- 35 ~ 40
- N.D.

Fig. S3. Cont.

E

Sample	Replicate	EAEC	EHEC	EIEC	EPEC (*bfpA*)	EPEC (*eaeA*)	ETEC (LT)	ETEC (STh/STp)	*L. monocytogenes*	*S. enterica* serovar *Typhimurium*
Cabbage Kimchi	1	26.50	30.30	30.46	30.89	30.21	32.10	30.66	34.09	32.30
	2	26.10	30.28	30.33	30.84	30.46	31.46	26.69	31.82	32.08
	3	22.89	29.36	29.89	30.94	30.11	31.22	28.55	33.30	32.82
Raddish Kimchi	1	25.36	30.17	30.42	31.02	29.98	30.52	24.33	32.45	32.40
	2	26.04	30.19	30.24	32.07	31.15	31.17	26.17	32.33	32.42
	3	26.55	29.46	29.69	30.88	31.23	30.61	24.32	31.69	31.82
Leaf mustard Kimchi	1	26.82	30.67	30.81	30.37	31.44	31.54	28.15	34.36	33.81
	2	27.42	29.70	30.69	30.01	30.90	31.45	28.81	33.28	33.00
	3	26.21	29.44	30.25	30.52	31.55	30.99	27.49	33.32	33.43
Greek yogurt	1	31.33	30.49	31.60	30.54	31.72	33.40	32.19	32.24	31.53
	2	31.10	30.43	31.19	29.47	31.02	32.54	31.04	34.11	33.27
	3	32.47	31.79	32.78	30.60	32.65	34.16	34.85	34.85	32.41
Yoghurt	1	33.27	32.04	33.29	33.03	33.76	33.22	33.11	35.16	33.97
	2	30.82	30.19	30.73	30.10	31.03	33.07	31.81	32.99	32.38
	3	31.59	30.77	31.91	29.89	32.52	34.52	31.79	33.96	32.84
Liquid yogurt	1	34.33	32.78	34.41	31.53	33.74	34.78	35.40	34.58	33.80
	2	32.68	31.42	33.24	30.98	33.50	34.12	33.17	35.36	34.04
	3	33.11	33.55	33.92	32.66	33.89	35.27	33.65	34.88	33.52

CT value

- 15 >
- 15 ~ 20
- 20 ~ 30
- 30 ~ 35
- 35 ~ 40
- N.D.
